# Analysis of Spatial Heterogeneity and Influencing Factors of Ecological Environment Quality in China’s North-South Transitional Zone

**DOI:** 10.3390/ijerph19042236

**Published:** 2022-02-16

**Authors:** Haoran Yin, Chaonan Chen, Qingdong Dong, Pingping Zhang, Quantong Chen, Lianqi Zhu

**Affiliations:** College of Geography and Environmental Science, Henan University, Kaifeng 475004, China; yjsyhr@163.com (H.Y.); 15736870141@163.com (C.C.); dongqd961103@126.com (Q.D.); zhangpp@henu.edu.cn (P.Z.); a13804817736@126.com (Q.C.)

**Keywords:** China’s North-South Transitional Zone, eco-environmental quality, remote sensing monitoring, regional policy coordination, climate change

## Abstract

The ecological environment is important for the natural disaster prevention of human society. The monitoring of ecological environment quality has far-reaching practical significance for the functional construction of ecosystem services and policy coordination. Based on Landsat 8 operational land image (OLI)/thermal infrared sensor (TIRS) remote sensing image data, this study selected the normalized vegetation (NDVI), tasseled cap transformation humidity (WI), bare soil (SI), construction index (NDSI), and land surface temperature (LST) indexes from the aspects of greenness, humidity, dryness, and heat. Using spatial principal component analysis (SPCA) and the remote sensing ecological index (RSEI) analyzed the spatial differentiation characteristics and influencing factors of the original remote sensing ecological index (RSEI_0_). The results showed that: (1) the overall RSEI average value of the Qinling-Daba Mountains in 2017 was 0.61, and the ecological environment quality was at a “Good” level. Greenness contributed the most to the comprehensive index of the area, and vegetation distribution had a significant impact on the ecological environment quality of the study area. Heat is a secondary impact, and it has an inhibitory effect on habitat quality; (2) the overall distribution of regional ecological environment quality was quite different, with the ecological environment quality level showing a decreasing trend from low to high altitude; RSEI_0_ spatial heterogeneity at the optimal scale of 2 km was the largest, and the nugget effect was 88% which indicated a high degree of spatial variability, mainly affected by structural factors; (3) Slope, relief amplitude, elevation, the proportion of high-vegetation area, proportion of construction land area, and average population density significantly impact the spatial differentiation of RSEI_0_. The explanatory powers of slope and relief amplitude were 56.1% and 65.3%, respectively, which were the main factors affecting the spatial differentiation of the ecological environment quality in high undulation. The results can provide important scientific support for ecological environment construction and ecological restoration in the study area.

## 1. Introduction

The ecological environment is a complex system formed by the interaction between natural and social factors. The ecological environment is a characteristic of the comprehensive performance of each element and its function, and it is also the resource and environmental basis for human survival and development [1]. No single environmental element can scientifically and objectively reflect the quality of the ecological environment [2,3]. Eco-environmental quality is one of the important types of ecosystem service functions, and maintaining the stability of its functions is crucial to ecological environmental protection and the integration of natural resources. In particular, the mountains, which characterize a unique geographical unit of the earth’s land surface, have a fragile ecological environment and are more sensitive to global changes. They are the “amplifiers” of global change signals. Changes in the quality of the mountain ecological environment have an important impact on the surrounding areas [4]. The potential factors affecting the ecological environment of mountainous areas are currently dominated by climate and topography. Their changes have varying degrees of impact on the quality of the ecological environment [5]. In addition, the mountain ecological environment is affected more by environmental problems such as soil erosion, forest degradation, and land desertification than other areas; the heterogeneity of the mountain itself and the negative interference by human beings have exacerbated the vulnerability of the ecological environment [6,7,8]. Therefore, studying the impact mechanism of mountain ecological and environment assessment is of great significance in constructing an ecological civilization and estimating the value of ecosystem services.

Satellite remote sensing technology has been widely used in ecological environment monitoring and environmental assessment in recent years, based on the high speed of remote sensing satellites and the macroscopic nature of real-time monitoring of the ground truth, and many scholars have used remote sensing indexes to assess the urban ecological environment [9,10,11], ecological demonstration area [12], aquatic environment [13], and terrestrial surface vegetation [14,15,16], and have obtained important theoretical results. However, the index selection is relatively single, mainly because the natural environment is a more complex ecological environment. The indicators selected for fragile areas lack scientific knowledge, and it is impossible to grasp the overall habitat quality from a macro perspective. Subsequently, the ecological environment evaluation index (EI) issued by the Ministry of Environmental Protection in 2006 has been widely used in China. Still, the determination of the weight value of the method and the setting and acquisition of the index is very simple [17], making it impossible to visualize the quality of the regional ecological environment. It cannot monitor the spatial change characteristics of the ecological environment any better [18]. Second, many studies are based on multi-angle evaluation methods, using comprehensive evaluation methods such as the fuzzy evaluation method and the analytic hierarchy process (AHP) to analyze the ecological environment and its quality standards; however, human subjectivity is strong, and it is widely used in research and cannot be sufficiently compared with regional differences. When studying the differences between regions, it is difficult to comprehensively evaluate the ecological environment quality using multiple composite factors [19,20]. Xu proposed a remote sensing technology-based regional, remote sensing ecological index (RSEI) in 2013 [11,21]. The calculation of this index is relatively convenient, and multiple environmental index factors are considered comprehensively, which can compensate for the suitability of the subjective analysis method for areas with more complex geographical environments. In recent years, it has been widely used by many scholars in the detection and evaluation of temporal and spatial changes in ecological environment quality [20,22,23,24,25]. However, systematic analysis of regional ecology research on the impact of environmental quality change and its driving factors is relatively scarce. The spatial differentiation characteristics of mountain ecological and environmental quality have rarely been studied. Therefore, the use of RSEI has important scientific and practical significance for research on the spatial difference of the mountain ecological environment quality at a mesoscale, which is conducive to an accurate understanding of the ecological environment problems in mountainous areas and for the better development of research.

The Qinling-Daba Mountains are the main components of China’s North-South Transitional Zone. The mountains are undulating and the vegetation coverage is relatively high. Abundant vegetation and water resources and diversified wild animal resources have created a complex ecological and geographical environment. As a natural barrier for ecological security in central China, an important ecological function area, its natural ecosystem is typicality and representativeness [26]. The quality of the ecological environment is critical. Current research is mostly based on areas such as southern Shaanxi [27], Taibai Mountain [28], and single administrative units [9], discussing the human factors of ecological environment degradation and the scientific nature of the regional ecological environment quality index system, based on the research of land use ecological risk assessment. There is no specific discussion on the quality of the ecological environment based on the Qinling-Daba Mountains as a whole, which leads to relatively imprecise research on the ecological environment of the area. Therefore, it is impossible to look at the trend as a whole. In addition, the study found that the analysis of different scale features and the selection of indicators are very important [29], considering that the Qinling-Daba Mountains are mesoscale regional mountainous landforms, the vegetation coverage is moderate, and the distribution of water and heat is different according to the terrain gradients. For this reason, the normalized vegetation index, the tasseled cap transformation humidity index, the bare soil index, the man-made building index, and the surface temperature were selected as the main factors to explore the spatial distribution of ecological quality based on remote sensing data and its impact mechanism. Finally, whether Structural factors or random factors affect the quality of the ecological environment, and how the local characteristics of the region lead to this process. Whether the research in this area can supplement the shortcomings and deficiencies of other scholars, and how it is different from other studies, are worthy of exploration. Therefore, this study uses the Qinling-Daba Mountains as the research area, based on the 2017 Landsat 8 OLI/TIRS remote sensing images, and uses the special principal component analysis method to construct the regional RSEI to comprehensively evaluate the ecological environment quality of the Qinling-Daba Mountains, analyze the spatial pattern and differentiation characteristics of the ecological environment quality, and systematically explore the potential factors affecting ecological and environmental quality. This study can explore a set of comprehensive remote sensing monitoring methods to monitor ecological environment changes in the areas. It aims to provide theoretical guidance and decision support for managers regarding soil erosion control, disaster prevention, and ecological environment restoration in the area and promote the sustainable development of the regional ecological environment.

## 2. Materials and Methods

### 2.1. Study Area

**Figure 1:**
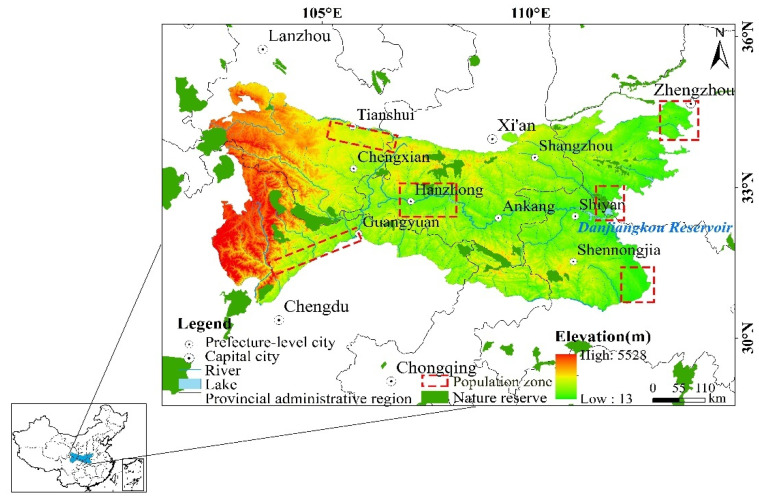
Geographical location of study area.

The study areas range from 30°–36° N and 101°–114° E to the east of the Qinghai-Tibet Plateau in the west, the eastern plain in the east, the southern edge of the Loess Plateau, and the northern Sichuan Basin, with the Qinling and Daba Mountains as the main body. It is approximately 300 km from north to south, with a span of nearly 1000 km from east to west, a total area of approximately 300,000 km^2^, covering the six provinces of Sichuan, Henan, Gansu, Shaanxi, Chongqing, and Hubei. The terrain slopes from west to east (Figure 1). The climate is dominated by the subtropical monsoon climate, high vegetation coverage, and rich biodiversity. It is in the transition zone between subtropical and warm temperate zones [30]. Affected by geographical location and mountain topography, the special area has a unique ecological and geographic environment, and has become an important ecological function area in the country. The average net primary productivity (NPP) of vegetation refers to the remainder after deducting autotrophic respiration from the total amount of organic dry matter produced by photosynthesis in a unit of time and area. It represents the vegetation growth status. The average net primary productivity of the areas has increased annually, forming an ecological environment pattern made of a multi-dimensional zonal landscape [31].

### 2.2. Data Source and Processing

**Figure 2:**
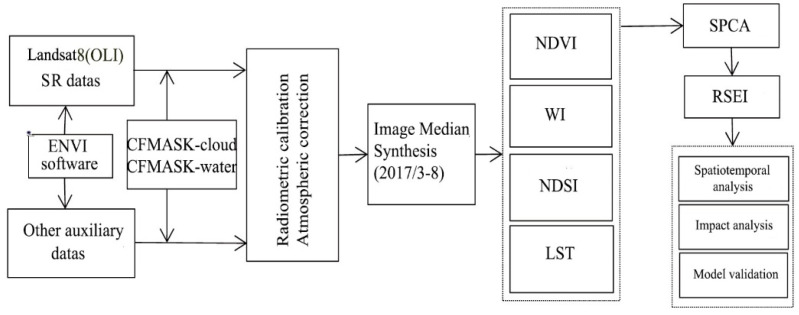
Technical roadmap. NDVI: normalized vegetation; WI: tasseled cap transformation humidity; NDSI: construction index; LST: land surface temperature; SPCA: spatial principal component analysis; RSEI: remote sensing ecological index.

The remote sensing data in this study area were obtained from the OLI image of the geospatial data cloud Landsat 8, with a spatial resolution of 30 m, involving 26 images of the study area from March to August 2017. The cloud cover of the images was less than 5%, and they were concentrated in the spring and summer, without falling leaves. The seasons are similar to each other, which can avoid the influence of seasonal differences. The overall workflow is shown in Figure 2.

**Figure 3:**
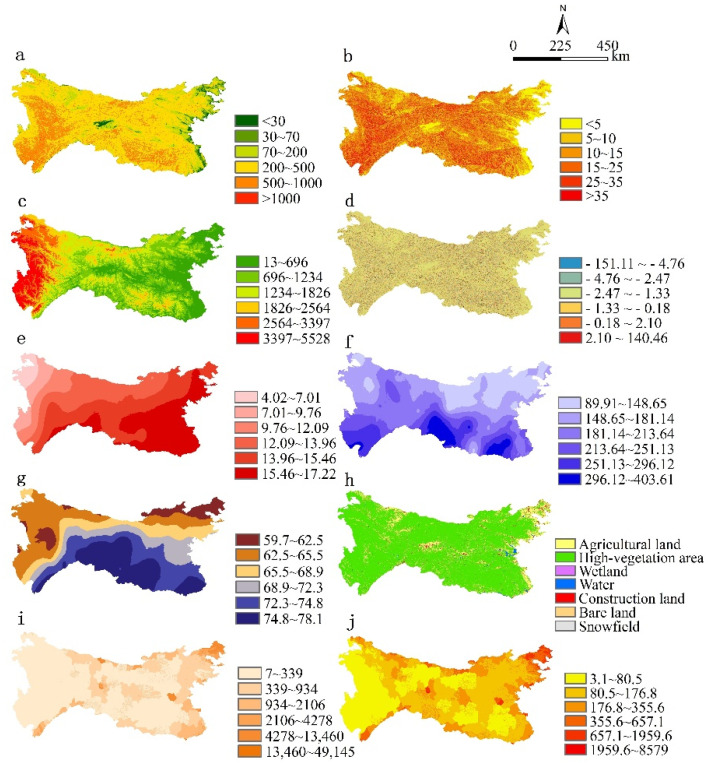
Distribution of each factor class of the Geodetector in Qinling-Daba Mountains. (**a**) Relief (m); (**b**) slope (°); (**c**) elevation (m); (**d**) curvature; (**e**) mean annual temperature (°C); (**f**) mean annual precipitation (mm); (**g**) mean annual relative humidity (%); (**h**) land use types; (**i**) mean annual GDP (yuan km^−2^); (**j**) Mean annual population density (person km^−2^).

The land cover data of the study area in 2017 comes from the FROM-GLC10 dataset developed by the Department of Earth System Science of Tsinghua University (http://data.ess.tsinghua.edu.cn) (accessed on 11 October 2020), with a spatial resolution of 10 m; the meteorological data is from China Meteorology. The daily value data of the Science Data Center (http://data.cma.cn) (accessed on 15 October 2020) are interpolated to obtain a raster dataset; the soil organic carbon data is in the Harmonized World Soil Database (HWSD) (http://www.fao.org/land-water/en) (accessed on 23 October 2020), the spatial resolution is 30 arc seconds, and the data of the entire study area is obtained by stitching and cutting; the soil erosion data of the study area are obtained according to previous studies [32]. The socio-economic data for 2017 is based on the 2015 annual average population and GDP, and is replaced by raster data, both from the Resource and Environment Data Center of the Chinese Academy of Sciences (http://www.resdc.cn) (accessed on 25 May 2020), with a resolution of 1 km; the DEM data comes from NASA_SRTM with a spatial resolution of 90 m. In the data set, the elevation, slope, and curvature-related data information are extracted. Combined with the actual situation of the study area, the average change-point method is used to extract the relief amplitude data of the areas. The input variables of the Geodetector are categorical, and the terrain relief amplitude is divided into six levels [33]: flat (<30 m), mesa (30–70 m), hills (70–200 m), small undulating mountains (200–500 m), medium undulating mountains (500–1000 m), and large undulating mountains (>1000 m). Other influencing variables are divided into six categories by the ArcGIS natural breakpoint method so that the results of the impact factors at all levels are comparable in the case of stratification. The results for each Geodetector category component are shown in Figure 3.

### 2.3. Methodology

#### 2.3.1. Selection of Various Index Factors of RSEI

This study selected the normalized vegetation index, changing the constitution of the tasseled cap, Bare soil index, Construction Index, and Surface Temperature from the perspectives of vegetation, soil, human activities, and climate [13,21]. Combining the spatial principal component analysis (SPCA) to construct the RSEI of the study area, the spatial differentiation of the ecological environment quality of the Qinling-Daba Mountains was assessed (Table 1) [34].

In this study, the NDVI, closely related to the leaf area index, surface vegetation, and biomass, is widely used to study vegetation and environmental changes as the greenness index [35].

Second, based on OLI land imager data, this study uses the tasseled cap change correlation coefficient method [36], combined with Gram–Schmidt and Prueck correlation algorithms, to obtain the study area atmospheric reflectivity remote sensing data. Regarding the OLI coefficient of variation method, the calculation results show that the accuracy is relatively high, and it has good sorting properties for ground objects [37]. It reflects the abundance of moisture in the soil, water bodies, and vegetation in the study area.

Third, to better characterize the degree of “Dryness” of natural and anthropogenic land in the study area, this study uses the bare soil index [38] and the building index [39] to indicate the dryness components. It takes the average value of the indexes for the quantitative calculation, which represents the natural area of the region and the ecological degradation caused by human activities.

Finally, the temperature component in this study is derived from the ground radiation ratio and the ground surface temperature corrected by the black body image grayscale, which can better quantify the energy exchange parameters between the surface image and the atmosphere and more accurately reflect the surface temperature. Therefore, using the atmospheric correction method to retrieve land surface temperature [40]. ε is estimated based on the NDVI and vegetation coverage image according to the Landsat model [41]. T was calculated using the Landsat model manual and the latest revised calibration parameter method [42,43]. It should be noted that, before calculating the blackbody radiance value, it is necessary to perform radiometric calibration and surface emissivity correction and select the Landsat 8 calculation formula to invert the ground temperature. We referred to the previous improved single-channel algorithm (SC algorithm) to verify and check the ground surface temperature [44]. The last three parameters mentioned above can be queried through the NASA website (http://atmcorr.gsfc.nasa.gov) (accessed on 3 October 2020) to obtain atmospheric profile parameter information.

#### 2.3.2. Construction of Comprehensive Index of Ecological Environment Quality Evaluation

In this study, the SPCA was used to couple the above four indicators to eliminate the high correlation between variables to reduce the inaccuracy of information caused by anthropogenic factors; the indicators were normalized through a unified dimension, and the initial study area was obtained. The initial RSEI_0_ was normalized again to obtain the final comprehensive index RSEI.

Positive normalization processing formula:

(1) $$N_{i}=\frac{M_{i}-I_{\min}}{I_{\max}-I_{\min}}$$

Reverse normalization processing formula:

(2) $$N_{i}=\frac{I_{\max}-M_{i}}{I_{\max}-I_{\min}}$$

The initial RSEI_0_ is obtained by principal component analysis:

(3) $${\mathrm{RSEI}}_{0}={\mathrm{PC}}_{1}\left[f\left(\mathrm{NDVI},\mathrm{WI},\mathrm{NDSI},\mathrm{LST}\right)\right]$$

Then normalize RSEI_0_:

(4) $$\mathrm{RSEI}=\frac{{\mathrm{RSEI}}_{0}-{\mathrm{RSEI}}_{0\min}}{{\mathrm{RSEI}}_{0\max}-{\mathrm{RSEI}}_{0\min}}$$

where M_i_ and N_i_ represent the pixel values of RSEI_0_ before and after normalization, respectively; I_max_ and I_min_ represent the maximum and minimum values of the image, respectively, and PC_1_ represents the first principal component of the four indicators.

#### 2.3.3. Spatial Heterogeneity Analysis Method of Ecological Environment Quality

Because natural and human factors have different degrees of impact on the environmental quality of mountainous areas, to distinguish the impact of structural factors and random factors on the ecological and environmental benefits of the areas and their structural degrees, this study uses a semi-variance function to analyze the degree of spatial differentiation and structural characteristics quantitatively. The image was gridded and sampled based on the optimal research scale of 2 km, and a 2 km scale spatial structure model was constructed. Based on whether the GS+9.0 conforms to the normal distribution and following the removal of a few sample points that do not meet the normal distribution, the square root conversion conforms to the normal distribution and meets the requirements of semi-variance function analysis. The semi-variance function formula is as follows:

(5) $$F\left(h\right)=\frac{1}{2I\left(h\right)}\overset{I\left(h\right)}{\underset{i=1}{\sum }}{\left[\left(W\left(C_{i}\right)-W\left(C_{i}+h\right)\right)\right]}^{2}$$

where F(h) is the value of the semi-variance function with h as the distance, h is the sample point spacing, I(h) is the number of sample pairs divided by the distance point, and W(C_i_) and W (C_i_ + h) are located in the range of C_i_ and C_i_ + h interval variables. The “Nugget effect” refers to the proportion of spatial autocorrelation heterogeneity at a scale. When C/C_0_ + C < 0.25, the spatial correlation is weak, indicating that the spatial variation caused by the random part plays a major role, and the spatial heterogeneity is weak. When C/C_0_ + C is between 0.25 and 0.75, the degree of correlation is moderate, and its spatial variability is determined by both random and structural factors. When C/C_0_ + C > 0.75, the spatial correlation is very strong, and its spatial variability is mainly caused by structural factors, and the degree of spatial heterogeneity is relatively large [45,46,47].

By combining the above sorting results, this study uses the factor detection of Geodetector to analyze the influence of each geographical element on the ecological environment of the Qinling-Daba Mountains to further study the independent interpretation of the influencing factors of the ecological environment quality to obtain the main factors affecting the differentiation of the mountain ecological environment quality. The formula for the influence degree of mass spatial heterogeneity is as follows:

(6) $$q=1-\frac{1}{{D\alpha }_{i}{}^{2}}\overset{n}{\underset{i=1}{\sum }}{\mathrm{Di}\alpha }_{i}^{2}$$

where q is the degree of influence of each influencing factor on the spatial distribution of the area’s ecological quality, q ∈ [0,1], D is the total number of samples in the study area, α_i_^2^ is the variance of the sub-regional ecological, environmental quality indicators, i = 1, 2... n, i represents each secondary partition, and n represents the number of all partitions. The size of q reflects the degree of spatial heterogeneity of the ecological environment quality in the area. The larger the value of q, the stronger the heterogeneity within the spatial partition. The various factors of the partition have a greater impact on the spatial distribution of the ecological environment quality and vice versa. In particular, when q = 0, it indicates no spatial heterogeneity in the ecological environment quality of the study area; when q = 1, it indicates perfect spatial heterogeneity. Geodetectors can perform a significant test for the q value [48].

#### 2.3.4. Eco-Environmental Quality Index (EQI) Verification

To verify how scientific and accurate the value of RSEI is in this study, EQI is combined with possible influencing factors, and principal component analysis is used to couple and analyze multiple environmental indicators [49], as follows:

(7) EQI=SPCA{f(TEM,PRE,TI,LAND,SL,SOM,POP,GDP)}

where TEM and PRE are the average annual temperature and annual precipitation in the study area, respectively, TI is the topographic index based on slope and altitude, LAND is the land use degree, SL is the soil erosion intensity, SOM is the soil organic matter content, and POP and GDP are the population density and total GDP, respectively.

## 3. Results

### 3.1. Principal Component Analysis Results of RSEI Index in Qinling-Daba Mountains

To comprehensively study the quality of the ecological environment of the Qinling-Daba Mountains in 2017, according to the research classification standard of Xu Hanqiu [21], the RSEI is divided into excellent (0.8–1.0), good (0.6–0.8), middle (0.4–0.6), poor (0.2–0.4), and very poor (0–0.2).

Principal component analysis was performed using the ENVI software to obtain the covariance matrix and correlation coefficient matrix of each component. The eigenvalues and contribution rates are presented in Table 2. It shows that the cumulative contribution rate of PC_1_ has reached more than 85%, indicating that it has concentrated on the main characteristics of the four indicators, and the first principal component can be used to replace the four indicators. The overall RSEI average of the Qinling-Daba Mountains reached 0.61, and the overall ecological environment quality was at a “good” level. It can be seen from the PC_1_ load values of the four component indicators that the greenness NDVI and humidity WI are positive in the overall load, and the dryness NDSI and heat LST are negative. Consistent with the fact that greenness and humidity positively affect the quality of the ecological environment, dryness and heat negatively affect the environmental quality [24,50,51,52]; the load value of PC_2_–PC_4_ can be of different sizes and cannot represent the overall component well. Among the positive indicators, the average value of NDVI was 0.63, and its contribution to the indicator was 0.614. It has the largest contribution of all indicators, suggesting that vegetation plays a vital role in the quality and maintenance of the ecological environment of the area. Humidity is second only to greenness in the ecological environment of the Qinling-Daba Mountains. The load value of PC_1_ was relatively low, whereas PC_2_ was relatively large and negative. Such findings contrast the large wetland in the area located in the urban concentration area with the low humidity in the high-altitude zone; humidity is affected by terrain and is man-made. Among the negative indicators, heat has a large negative impact on the ecological environment quality of the overall study area, and loads of PC_1_ and PC_2_ are opposite, indicating that appropriate temperature and light have a positive effect on vegetation and biological growth. However, affected by geomorphic features and aspects, vegetation and soil moisture will evaporate faster, and the habitat benefit will gradually decline.

### 3.2. The Spatial Distribution Characteristics of RSEI in Qinling-Daba Mountain Area

**Figure 4:**
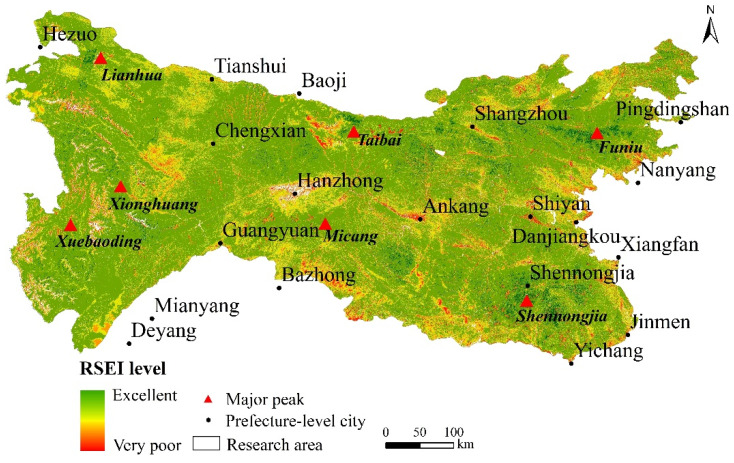
Spatial distribution of eco-environmental quality in Qinling-Daba Mountains by remote sensing.

It can be seen from Figure 4 that the ecological environment quality of the Qinling-Daba Mountains in 2017 is significantly different: excellent areas are scattered in the Shennongjia forest area, western Henan Mountains, Taibai Mountain, and western high-altitude areas, covering Shennongjia, Taibai Mountain, Baotianman, Funiu Mountain nature reserves, and Lianhuashan in Gansu. They have good natural vegetation growth and high ecological environment quality. The poor areas are mainly distributed in the middle and low altitude areas in the northwestern part of the west. The central Hanzhong area, and the areas along the eastern line, with relatively concentrated areas, are generally located in the fringe areas of cities, such as Hanzhong City, Danjiangkou City, Shiyan City, Nanyang City, and other important urban areas.

Table 3 shows the area and proportion of each quality grade of the ecological environment in the study area (water area was not included). Among the various grades of the area ecological environment quality, good (0.6–0.8) and medium (0.4–0.6) are 17.9 × 10^4^ km^2^ (63.14%) and 8.2 × 10^4^ km^2^ (29.15%), respectively, occupying a large proportion. Poor (0.0–0.2), very poor (0.2–0.4), and excellent (0.8–1.0) are 0.3 × 10^4^ km^2^ (1.11%), 1.3 × 10^4^ km^2^ (4.54%), and 0.58 × 10^4^ km^2^ (2.06%), respectively, accounting for relatively less. The overall distribution characteristics are “small at both ends and big in the middle,” and the proportion of excellent-grade and poor-grade areas is relatively small. In contrast, the area of middle-grade areas is larger. According to the characteristics of the terrain, the ecological environment quality of the middle and high altitude (1000–4500 m) mountainous areas are mainly excellent-grade and middle-grade areas, and the low-altitude (<1000 m) areas are mainly poor or very poor-level areas. The ecological environment quality level shows a decreasing trend from low to high altitude.

### 3.3. The Spatial Differentiation Characteristics of RSEI_0_ under Different Scale Effects

**Figure 5:**
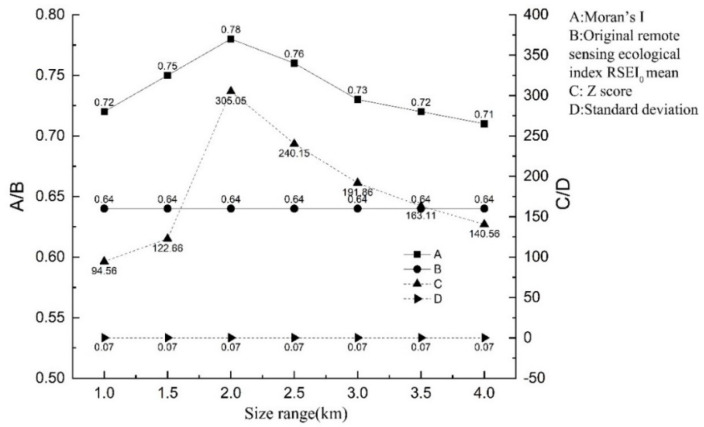
Spatial statistical characteristics of RSEI_0_ in different scales of Qinling-Daba Mountains.

It can be seen from Figure 5 that the Moran’s I shows a single-peak trend, first increasing sharply, before slowly decreasing, with the lowest value being 0.71; at the spatial scale of 1 × 1 km, 1.5 × 1.5 km, 2 × 2 km, 2.5 × 2.5 km, 3 × 3 km, 3.5 × 3.5 km, and 4 × 4 km, the values are 0.72, 0.75, 0.78, 0.76, 0.73, 0.72, and 0.71, respectively. The Z-score feature trend was similar to Moran’s I. All passed the significance test (Z-score > 1.96), indicating that RSEI_0_ has a strong positive spatial correlation at various scales and the spatial heterogeneity is relatively large. Second, the mean and standard deviation of RSEI_0_ under the seven scales did not change with the scale. The scale had a relatively small impact on the ecological and environmental benefits itself, thus avoiding the inequality of research objects at different spatial scales. The Moran’s I reach a maximum at 2 km of the grid, indicating that RSEI_0_ has strong spatial aggregation and spatial heterogeneity at this scale. The spatial distribution characteristics of RSEI_0_ are related to geographical locations so that RSEI_0_ can study spatial structure characteristics and factor detection.

Table 4 shows that the optimal fitting theoretical model of RSEI_0_ is the exponential model, the coefficient of determination (R-Square) is 0.80, the residual error (RSS) is 2.3 × 10^−7^, and its nugget effect is 88%, indicating that a high degree of space heterogeneity is mainly affected by structural factors. Among them, the nugget value (C_0_) of RSEI_0_ in the study area is relatively small (7.6 × 10^−4^), indicating that factors affect the spatial distribution of ecological environment quality on a small sampling scale. At the same time, the nugget effect (C/C_0_ + C) exceeded 0.75, indicating that the spatial heterogeneity dominated by structural factors was relatively large, and the randomness was relatively small. Combined with the Geodetector analysis results, the detection factors of elevation, slope, and relief amplitude were 0.528, 0.561, and 0.653, respectively, all of which passed the two-tailed significance test, which has a greater impact than climate (0.256, 0.312, and 0.23) and socio-economic (0.002, 0.174, and 0.214) factors (from Section 3.4). Such findings are due to the vast mountainous area in the study area. Large topographical undulations account for more than 1/5 of the total area, and they are mostly distributed in man-made protected nature reserves and mountain hinterlands. Topographic factors have a significant impact on the quality of the ecological environment. On the best analysis scale, the spatial differentiation of ecological environment quality in different regions is equally large, but it is worth noting that this region is a concentrated area for poverty alleviation under many plains, where urbanization and economic development are relatively slow. Because of the impact of key local engineering constructions such as returning farmland to forests and water and soil conservation in the areas, the ecological environment of the middle and low altitude areas is better than other areas in China, but it is different from other research results such as plateau, arid, and agricultural and pastoral areas [53,54,55,56]. Differences and comparisons will be described in the discussion section.

### 3.4. Factors Influencing Spatial Heterogeneity of RSEI_0_ in Qinling-Daba Mountains

In this study, the Pearson correlation analysis method was used to analyze the correlation between each variable. As shown in Table 5, the RSEI_0_ in the study area positively correlates with the slope, relief amplitude, elevation, and curvature in the terrain factors, with correlation coefficients of 0.62, 0.56, 0.52, and 0.1, respectively. The slope has the largest contribution rate to the terrain factor, followed by relief amplitude and elevation, indicating that the greater the slope, the higher the elevation, and the greater the relief amplitude, the better the quality of the ecological environment. Conversely, the lower the elevation, the lower the quality of the ecological environment. Among climatic elements, RSEI_0_ was negatively correlated with annual average temperature and annual precipitation, but positively correlated with relative humidity, consistent with the negative effect of heat on the quality of the ecological environment in the previous article (from Section 3.1). The annual precipitation correlation coefficient was 0.38, and that of the annual average relative humidity was 0.18. Although it is consistent with the PC_1_ load value and the positive correlation with RSEI_0_, the precipitation and humidity index correlation are different. This difference is because the mask image was extracted from the water area, and the most important wetland areas were removed, so the correlation between humidity and RSEI_0_ was weakened. Among the land use types, the correlation coefficients of the proportion of high-vegetation area, construction land, and agricultural land were 0.37, 0.33, and 0.09, respectively. Among them, only the proportion of high-vegetation areas had a high positive correlation with RSEI_0_. The ratio of construction land to agricultural land area is negatively correlated with the quality of the ecological environment, proving that the vegetation areas with higher altitudes mentioned above contribute to the quality of the ecological environment. At the same time, improving the ecological deterioration with high vegetation coverage has a positive effect on the quality of the ecological environment. The expansion of industrial agriculture in low-altitude areas has a restraining effect on the ecological environment of the study area. Among the socio-economic factors, the correlation coefficients between the population density and the average annual GDP are only 0.22 and 0.19, respectively. Both have a negative correlation with RSEI_0_, indicating that socio-economic development and human activities negatively affect the quality of the regional ecological environment. In summary, slope, elevation, relief amplitude, average annual precipitation, and high-vegetation land use types have a close spatial correlation with RSEI_0_, which affects the degree of spatial heterogeneity of the ecological environment quality.

**Figure 6:**
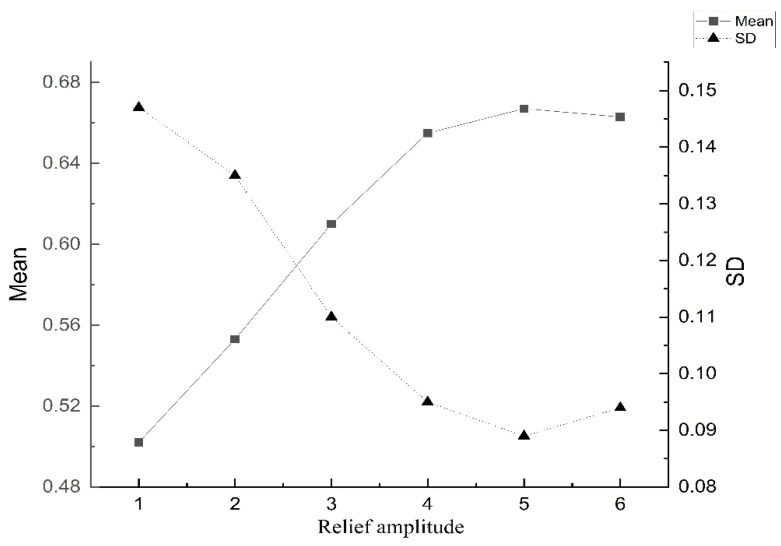
Distribution of mean value of RSEI_0_ under different relief amplitudes.

The effect of the influencing factors mentioned above was analyzed by exploring the explanatory power of each influencing factor on the spatial variability of RSEI_0_ in the Qinling-Daba Mountains based on geographic detectors. As shown in Table 6, slope, elevation, curvature, relief amplitude, annual average temperature, annual average precipitation, annual relative humidity, the proportion of high vegetation area, the proportion of construction land area, and population density affect the ecological environment quality of the study area. The impact of spatial differentiation is more significant, but its explanatory power is different. Among the terrain factors, the interpretation ability of topographic undulation was the highest, reaching 65.3%; the second was elevation and slope, which were 52.8% and 56.1%, respectively; the weakest interpretation ability was curvature, which was only 2.1%. Among the climatic factors, the interpretation ability of the spatial differentiation of annual average precipitation is higher (31.2%); the annual average temperature and relative humidity are second, only 25.6% and 23%, respectively. Among the land use types, high-vegetation areas accounted for a higher explanation ability (28.2%), followed by construction land (13.5%). Among the socio-economic indicators, the explanatory ability of population density was 21.4%. The spatial heterogeneity of the relief amplitude was relatively high, exceeding 60%. As shown in Figure 6, the average value of RSEI_0_ in the area increases with the relief amplitude on flat land (<30 m), mesa (30–70 m), hills (70–200 m), and small relief mountains (200–500 m). However, the sharp increase began slowly after the medium-relief mountain area (500–1000 m). The change also quickly stabilized, indicating that the overall ecological quality can be quickly improved through adjustment and optimization measures in the medium and small relief mountain areas. The high relief mountain area needs to maintain the current good ecological environment by maintaining the water, soil, and vegetation. In summary, structural factors have a greater impact on the RSEI_0_ of the Qinling-Daba Mountains. Among them, the spatial differentiation of relief amplitude has the highest interpretation ability. The influence of slope on RSEI_0_ cannot be underestimated and is consistent with the results of the semi-variance function study. Such results show that the quality of the ecological environment in the study area is restricted by natural factors. The influence of social and economic factors is not enough to offset the changes in the regional mountain ecological environment. The relief amplitude and slope factors can be considered to improve the ecological environment quality of the entire region in the future.

## 4. Discussion

### 4.1. Verification of the Accuracy of the RSEI Comprehensive Method

**Figure 7:**
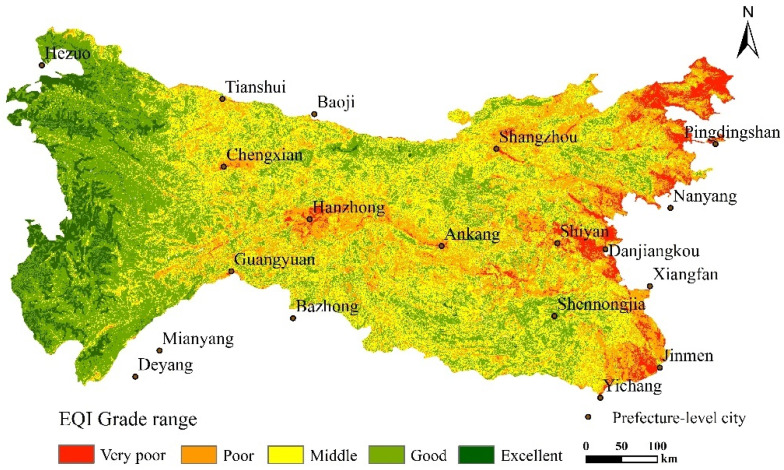
Spatial distribution of ecological environment quality index (EQI).

This study uses RSEI’s comprehensive analysis index to characterize the ecological environment quality of the Qinling-Daba Mountains and analyzes the spatial structure differentiation characteristics and its influencing factors. The advantage of using this method is that a comprehensive index can quantitatively analyze the objects referred to by the index through the linear transformation of the data itself. The transformation algorithm is not obtained by an artificially weighted calculation, which overcomes the subjectivity of artificial evaluation. Second, the whole area has the characteristics of “population gathering” and has different levels of human activities in the complex natural geographical environment [32]. The simple and superimposed ecological environment evaluation method cannot be visualized, and the weight of a single element is relatively large. The method proposed by the present study overcomes this drawback. The analysis results show that the average values of the humidity index, dryness index, and heat index are 0.07, 0.21, and 0.03, respectively, and are less than the comprehensive index RSEI. The greenness index with the highest contribution is 0.2, which is higher than that of RSEI. The correlation between the indexes is also very high. These values show that this comprehensive index can better integrate the information of each index and is also more representative than any single index, and can adequately reflect the quality of the ecological environment in the area. The EQI also verified the overall spatial distribution trend [49], which is highly fit with RSEI. However, RSEI is better than the verification data in local details, indicating that this comprehensive indicator is suitable for detecting the Qinling-Daba Mountains’ ecological environment (Figure 7).

### 4.2. The Rationality of the Selection of Indicators

In addition, the selection of indicators is scientific and reasonable, affecting the distribution of comprehensive indicators. The NDVI index selected for greenness in this study was due to the large differences in vertical relief in the Qinling-Daba Mountains areas, the longer plant growth seasons under different terrains, and the medium-level vegetation coverage that contributes to the ecological environment; therefore, the use of this index can reduce the detailed problem of special principal component analysis and is suitable for studying the mesoscale area [29,57]. The selection of humidity and heat indicators mainly consider the complexity of the areas’ climate and the characteristics of the mountain elevation effect mechanism that led to a large difference in heat distribution between the mountain and the plain [58]. This selection is reflected by the differences in the internal and external environments of the mountains, resulting in huge differences in the distribution of humidity and heat, which indirectly affects the quality of the regional ecological environment through biological effects [59]. The selection of dryness includes unused natural wasteland and man-made construction land. Frequent human activities in low-altitude areas in the mountain area and the loss of natural wasteland in high-altitude areas strongly antagonistically affect ecological quality. This indicator takes natural and human factors into account and can be compared with the overall environmental quality, and comprehensively analyzes the spatial differences in ecological, environmental quality.

### 4.3. Comparative Analysis and Suggestions on Influencing Factors

Structural factors have a more important impact on the ecological environment quality of the Qinling-Daba Mountains than random factors, especially the spatial interpretation of relief amplitude and slope, which can reflect the spatial differentiation of RSEI more than other factors. The change characteristics are mainly due to the diverse landform types of the area, and the complex geographic features make the spatial geographic differentiation huge [60]. Vegetation coverage is densely distributed in areas with large relief amplitudes and slopes due to fewer human activities; areas with lower undulations are subject to frequent human disturbances. The ecological environment is restricted by construction and agricultural land.

At the same time, we found that the explanatory power of the annual average precipitation was 31.2%. In comparison, the explanatory power of the agricultural land area was 0.2%, with a weak negative correlation, which is in accordance with similar studies [23,55]. In their results, the ecological environment of agricultural areas was relatively high, and the positive correlation was inconsistent. Such findings are due to intensive and unreasonable farming methods in the lowland plains of the area that hinder the development of the regional ecological environment. We also believe that there are various problems in the regional agricultural ecological environment, including soil pollution caused by chemicals such as pesticides, water pollution caused by breeding and domestic sewage, and backward energy utilization patterns that lead to energy waste and air pollution. In addition, the study time was selected as the spring and summer season, which is the farming season, when the vicinity of the original vegetation environment is affected by farming. These factors led to a weak negative correlation between the quality of the ecological environment and agricultural land.

On the other hand, the explanatory power of annual average temperature, annual average GDP, and population density are 25.6%, 17.4%, and 21.4%, respectively, and are negatively correlated with RSEI_0_, which is more consistent with the results of previous studies [52,61,62]. In view of this result, we all agree that the Qinling-Daba Mountains are one of the most sensitive areas to climate change. The climate varies greatly in some mountainous areas, and the temperature difference is more obvious. Abrupt climate change has a certain impact on the growth of vegetation in the transition zone, especially the increasing trend of extreme and high-temperature weather and the difference in water and heat evaporation caused by slope direction [63], combined with regional warming to reduce the quality of regional habitats to a certain extent. As far as socioeconomic indicators (annual average GDP, population density) are concerned, because the Qinling-Daba Mountains area is one area where the poor population is concentrated in China, various social and economic development indicators are relatively weak [64]. The lack of coordination between the natural ecological environment and economic development makes the ecological environment subject to less human disturbance. It maintains a virtuous cycle of the ecosystem in areas with relatively poor economic development [65,66,67,68].

At the same time, we also found that the low ecological, environmental quality areas are located near many cities; the expansion of local land scale, infiltration of human activities, unreasonable land planning, the pursuit of the formal economy, and other adverse social reactions have negatively affected the nearby ecological environment. The large areas are located in the transition zone from the northern subtropical to the warm temperate zone [31]. The ecological environment is relatively fragile and sensitive. Although the implementation of many environmental measures has effectively improved the resilience of the mountain ecological environment, the area is suffering in terms of special ecological environment construction. Therefore, we must coordinate the overall situation and pay more attention to the local, regional governance of mountain characteristics, water and soil, and man-made environments.

### 4.4. Shortcomings and Prospects

The disadvantage of this study is that it does not fully explore the deeper index framework of the ecological environment. It only analyzes the quality of the ecological environment on the land surface system without considering the influence of water bodies and human ecological environment index factors. The time phase of remote sensing data does not reach a very high degree of closeness, and there is a certain error in the results of the analysis of RSEI. Moreover, climate change has an important impact on the quality of ecosystems and the ecological environment and should be considered in the future [69,70]. Limited by the number of data acquisitions, this study only studies the 2017 Qinling-Daba Mountains’ RSEI and its influencing factors and has not carried out multi-dimensional studies on multiple years to reveal its temporal and spatial distribution characteristics. In the future, the quality of the ecological environment in different regions of the Qinling-Daba Mountains will be studied on a long-term scale, including research on topography, extreme climate, atmospheric circulation, water body ecological environment, and human effects. The research is mainly based on high-precision spatial resolution remote sensing image data to explore the ecological status of different provinces and urban areas, and to reveal the impact mechanism of atmospheric changes and human activities on the quality of the ecological environment. Future work will continue to advance this research.

## 5. Conclusions

(1) The overall RSEI average value of the Qinling-Daba Mountains reached 0.61, and the ecological environment quality was mostly above the middle level; the greenness contributed the most to the RSEI comprehensive index of the areas, indicating that vegetation coverage plays an important role in the improvement of the ecological environment quality of the areas. Heat has the second-highest contribution to the RSEI index of the area, and it has an inhibitory effect on improving the area’s habitat quality.
(2) The overall distribution of ecological environment quality in the study area in 2017 was quite different, with good and bad being distributed alternately from east to west; the ecological environment quality level decreased from low to high altitude. Low-value areas accounted for a relatively large area in low-altitude land, and high-value areas accounted for a relatively large area in high-altitude areas.
(3) There are scale changes in the spatial clustering of RSEI_0_. The degree of spatial heterogeneity is the most obvious at a scale of 2 km. The RSEI_0_ nugget effect is 88%, which is high spatial heterogeneity, mainly affected by structural factors such as slope, relief amplitude, elevation, curvature, annual average temperature, annual average precipitation, annual average relative humidity, the proportion of high-vegetation areas, proportion of construction land area, and average annual population density which have significant effects on the spatial differentiation of RSEI_0_. Among them, slope and relief amplitude are the main factors affecting the spatial differentiation of the Qinling-Daba Mountains’ ecological environment quality.

## Figures and Tables

**Table 1:** RSEI evaluation index and its calculation formula.

Index	Calculation Formula
Greenness	NDVI = (NR − NIR)/(NR + NIR)
Humidity	WI = 0.1511B + 0.1973G + 0.3283NR + 0.3407NIR − 0.7117M_1_ − 0.4559M_2_
Bare soil and construction	NDSI = (SI + NDIBI)/2
	SI = [(M_1_ + NR) − (NIR + B)]/[(M_2_ + NR) + (NIR + B)]
	NDIBI = {2M_1_/(M_1_ + NIR) [NIR/(NIR + NR) + G/(G + M_1_)]}/{2M_2_/(M_2_ + NIR) +
	[NIR/(NIR + NR) + G/(G + M_2_)]}
Land surface temperature	LST = T/[1 + (λT/ρ) lnε] − 273
	T = B_2_/ln (B_1_/H_t_ + 1)
	H_t_ = (L_t_ − ↑U − V (1 − ε) ↓D)/V_ε_

Note: B, G, NR, NIR, M_1_, M_2,_ T, K, ρ, λ, ε, H_t,_, B_1_, B_2_, L_t_, ↑U, ↓D, V represent the reflectance of the Landsat 8 remote sensing image in bands 2, 3, 4, 5, 6, and 7, the surface brightness temperature (K = 1.38 × 10 − 23 J·K^−1^), ρ = 1.438 × 10 − 2 M·K(M is the default constant parameter set by the platform),the center wavelength of the OLI thermal infrared band (λ = 11.45 μm), the surface emissivity image (ε), the radiation value of the pixel in thermal infrared 10 band at the sensor, the calibration parameters (B_1_, B_2_), the thermal infrared band radiance image (L_t_), upward radiance value and downward radiance value (↑U = 1.64 W/(m^2^·sr·μm), ↓D = 2.75 W/(m^2^·sr·μm)), atmospheric profile thermal infrared transmittance (V = 0.78).

**Table 2:** Results of RSEI and its indicators.

Index	Mean	Standard Deviation	PC_1_	PC_2_	PC_3_	PC_4_	PC_1_ Load Value
NDVI	0.63	0.27	0.614	0.133	0.427	0.654	0.614
WI	0.54	0.16	0.232	−0.971	0.233	0.048	0.232
NDSI	0.40	0.20	−0.521	−0.194	−0.826	0.094	−0.521
LST	0.58	0.29	−0.597	0.030	0.285	−0.749	−0.597
Eigenvalues	-	-	0.193	0.023	0.005	0.004	-
Eigenvalue Contribution rate (%)	-	-	85.41	10.29	2.31	1.99	-
RSEI	0.61	0.10	-	-	-	-	-

**Table 3:** Area and proportion of remote sensing eco-environmental quality.

RSEI Level	Area (km^2^)	Proportion (%)
Very poor (0~0.2)	3154.5	1.11
Poor (0.2~0.4)	12,851.15	4.54
Middle (0.4~0.6)	82,487.02	29.15
Good (0.6~0.8)	178,672.98	63.14
Excellent (0.8~1.0)	5832.89	2.06
Total	282,998.54	100.00

**Table 4:** RSEI_0_ Semi-variance function model and its results.

Model	C_0_	C_0_ + C	C/C_0_ + C	R-Square	RSS
Gaussian model	0.001	0.0063	83.9	0.74	2.9 × 10^−7^
Linear model	0.002	0.0071	71.0	0.34	1.7 × 10^−7^
Exponential model	0.00076	0.0064	88.0	0.80	2.3 × 10^−7^
Spherical model	0.00032	0.0060	94.9	0.74	2.9 × 10^−7^

**Table 5:** RSEI_0_ and the correlation analysis result of each index factor.

Influencing Factor	Analytic Index	Correlation Coefficient	Correlation
Terrain factors	Elevation	0.52	+
	Slope	0.62	+
	Curvature	0.10	+
	Relief amplitude	0.56	+
Climatic factors	Average annual temperature	0.25	−
	Average annual precipitation	0.38	−
	Annual average relative humidity	0.18	+
Land use type	Proportion of high vegetation area	0.37	+
	Proportion of construction land area	0.33	−
	Proportion of agricultural land area	0.09	−
Socio-economic factors	Annual average GDP	0.19	−
	Population density	0.22	−

+: positive; −: negative.

**Table 6:** RSEI_0_ detection results of various influencing factors.

Factor Types	Influencing Factor	Detection Index	(q Value)
Structural factors	Terrain factors	Elevation	0.528 **
		Slope	0.561 **
		Curvature	0.021 **
		Relief amplitude	0.653 **
	Climatic factors	Annual average temperature	0.256 **
		Annual average precipitation	0.312 **
		Annual average relative humidity	0.230 **
Randomness factors	Land use type	Proportion of high vegetation area	0.282 **
		Proportion of construction land area	0.135 **
		Proportion of agricultural land area	0.002
	Socio-economic factors	Annual average GDP	0.174
		Population density	0.214 **

** *p* < 0.01.
